# FAITH-BASED COMMUNITY OUTREACH TO INCREASE ALZHEIMER’S CLINICAL RESEARCH PARTICIPATION IN THE BLACK COMMUNITY

**DOI:** 10.1093/geroni/igad104.3346

**Published:** 2023-12-21

**Authors:** Ethlyn Gibson, Bahar Niknejad, Hamid Okhravi, Ebony Andrews, Travonia Brown-Hughes

**Affiliations:** Norfolk State University - School of Nursing, Yorktown, Virginia, United States; Eastern Virginia Medical School, Norfolk, Virginia, United States; Eastern Virginia Medical School, Norfolk, Virginia, United States; Hampton University, Hampton, Virginia, United States; Hampton University, Hampton, Virginia, United States

## Abstract

Issues: Despite the fact that African Americans are twice more likely to develop Alzheimer’s disease (AD) compared to non-Hispanic whites, they are under-represented in AD clinical research. Lower representation exists in the AHEAD Study as well, which is an AD prevention clinical trial evaluating the efficacy and safety of Lecanemab in 1400 study participants. We sought to raise awareness about AD and increase clinical research participation of African Americans in the AHEAD Study, through a community-based education and recruitment program (CERP). Description: Our diverse team from Eastern Virginia Medical School and Hampton University, a Historically Black College University (HBCU), established a Community Advisory Board and Ministerial Alliance with representation from faith-based, long-standing civic and fraternal organizations within the Greater Hampton Roads Black community. The alliance guided us to identify prioritization areas for addressing sociocultural barriers and increasing clinical research awareness and participation in traditional African American events, key historic community sites, and non-traditional community centers (i.e., YMCA, independent-living, assisted-living centers, Annual Juneteenth Celebration, Black fraternity, and sorority events.) Community health workers, embedded within the community, of the same cultural background and ethnicity, drove our CERP. Lessons Learned: As of July 2023, we offered our program at 45 community events, including community health fairs, civics events, and Purple Sunday luncheons and talks at Black churches in urban and localities in Hampton Roads, in partnership with fraternal and civic community organizations. Out of over 1700 participants, 177 community members have been prescreened for the AHEAD Study.

